# Revisiting Antibiotic Resistance: Mechanistic Foundations to Evolutionary Outlook

**DOI:** 10.3390/antibiotics11010040

**Published:** 2021-12-30

**Authors:** Chowdhury M. Hasan, Debprasad Dutta, An N. T. Nguyen

**Affiliations:** 1 School of Biological Sciences, University of Queensland, Brisbane 4072, Australia; 2 Department of Clinical Infection, Microbiology and Immunology, Institute of Infection, Veterinary & Ecological Sciences (IVES), University of Liverpool, Liverpool L7 3EA, UK; debdutta.bio@gmail.com; 3 School of Biological Sciences, Monash University, Melbourne 3800, Australia; an.nguyen2@monash.edu; 4 Department of Human Genetics, National Institute of Mental Health & Neurosciences (NIMHANS), Bangalore 560029, India

**Keywords:** antibiotic resistance, bacteriostatic, bactericide, evolution, adaptation, epistasis, drug interaction, mutant selection window, compensatory evolution, clonal interference

## Abstract

Antibiotics are the pivotal pillar of contemporary healthcare and have contributed towards its advancement over the decades. Antibiotic resistance emerged as a critical warning to public wellbeing because of unsuccessful management efforts. Resistance is a natural adaptive tool that offers selection pressure to bacteria, and hence cannot be stopped entirely but rather be slowed down. Antibiotic resistance mutations mostly diminish bacterial reproductive fitness in an environment without antibiotics; however, a fraction of resistant populations ‘accidentally’ emerge as the fittest and thrive in a specific environmental condition, thus favouring the origin of a successful resistant clone. Therefore, despite the time-to-time amendment of treatment regimens, antibiotic resistance has evolved relentlessly. According to the World Health Organization (WHO), we are rapidly approaching a ‘post-antibiotic’ era. The knowledge gap about antibiotic resistance and room for progress is evident and unified combating strategies to mitigate the inadvertent trends of resistance seem to be lacking. Hence, a comprehensive understanding of the genetic and evolutionary foundations of antibiotic resistance will be efficacious to implement policies to force-stop the emergence of resistant bacteria and treat already emerged ones. Prediction of possible evolutionary lineages of resistant bacteria could offer an unswerving impact in precision medicine. In this review, we will discuss the key molecular mechanisms of resistance development in clinical settings and their spontaneous evolution.

## 1. Introduction

The introduction of antimicrobials to treat infectious diseases was one of the greatest medical achievements in history [1]. The objective was to save millions of lives facing severe infectious diseases caused by various pathogens, including bacteria, fungus, viruses, and parasites. Though these antimicrobial drugs have saved millions of lives, the global emergence of resistance mechanisms among these pathogens has seriously undermined our current treatment options [2,3]. Therefore, treatment failure due to the emergence of antimicrobial resistance is now a global public health threat and a tremendous economic risk [4,5]. Among these pathogens, bacteria are the most striking example in terms of morbidity and mortality. The worrying picture is that by 2050 global death due to an increase in antibiotic resistant bacteria is estimated to be 50 million per year at an increasing global cost of $100 trillion USD [6]. Bacteria exploit various efficient strategies to neutralize the action of antibiotics, often leaving no effective treatment options that can be employed to treat infectious diseases caused by them. It is well evident that bacteria employ various physiological and biochemical mechanisms to develop tolerance or resistance [7]. This review will highlight types of antibiotics, their mechanisms of action, and the mechanistic and evolutionary basis underlying resistance evolution in bacterial populations.

## 2. Antibiotics and Their Impact on Bacterial Cellular Perturbation

**Figure 1:**
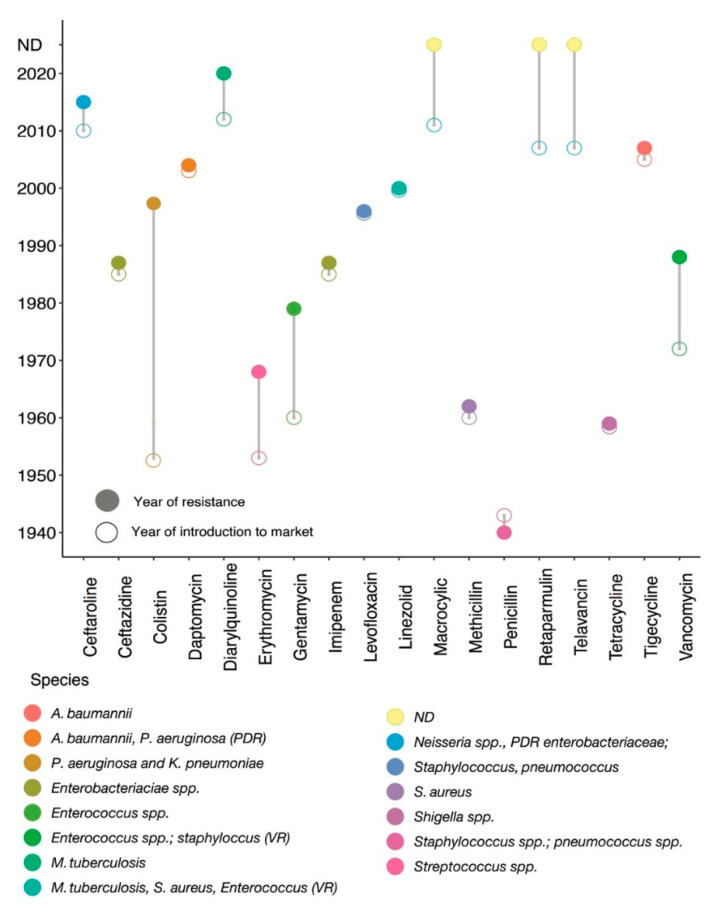
Historical panorama of antibiotic launch and resistance detection. The x-axis indicates different types of antibiotics and the corresponding y-axis shows the year of introduction into clinical practices. Resistance histories to different antibiotics are shown by different circles. Connecting line between empty and filled coloured circles shows the year of introduction of a specific antibiotic into clinical practice and the year of resistance observed for that antibiotic; each coloured circle further represents different bacterial species. For example, colistin was first introduced into clinical practice in 1952 [13], but resistance to colistin was first reported in clinical *P. aeruginosa* and *K. pneumoniae* (shown by a specific coloured circle) in 1998 [14]. Penicillin resistant laboratory *E. coli* was reported in 1950 [15] before its introduction into clinical practice in 1941, but the first penicillin resistance clinical *S. aureus* was reported in 1942 [16,17]. PDR: pan-drug resistant; VR: vancomycin resistant; spp: species; ND: resistance mechanism not detected.

The discovery of antibiotics has drastically changed modern medicine and extended the human lifespan. The first naturally occurring antibiotic, namely penicillin, was discovered by Alexander Fleming from *Penicillium notatum* in 1928, which was introduced into clinical practice in 1941 [8,9]. Most of the antibiotics that are used today were discovered and introduced into the market before 1987, and this period is often termed as the golden decade [10]. Particularly, the cessation of new antimicrobial classes was apparent from 1987 until today [11]; during this period, only a few (~9) new antimicrobial classes were discovered and launched into the markets (Figure 1). With this slow rate of new antibiotic discovery and the rapid emergence of resistant bacteria, we are in the post-antibiotic era [12].

Antibiotics belong to different classes of chemicals—including those of biological, synthetic, or semi-synthetic origin—and have selective modes of action. Based on their mechanisms of action, antimicrobial compounds are classified into two groups: bacteriostatic (Table 1) and bactericidal (Table 2). Bacteriostatic drugs are only able to inhibit or hinder growth but cannot kill bacteria. Whereas drugs are called bactericidal when exposure to this group of antibacterial compounds leads to the death of bacteria. Both drugs principally interfere with bacterial cell-wall biosynthesis, DNA synthesis, RNA synthesis, and protein synthesis [18]. Furthermore, antibiotics have been classified based on the cellular components or systems they affect in bacteria. Some of these antimicrobial agents target the synthesis of important cellular components, whereas some other classes of antibiotics interfere with bacterial nucleic acid synthesis or repair [19,20]. The mechanistic actions of the bacteriostatic and bactericidal are featured in Table 1 and Table 2. Furthermore, we have described the mechanistic action of the major bactericidal antibiotics in the following sections.

A commonly used bactericidal class of antibiotic is fluoroquinolone which targets and inhibits DNA replication by interfering with topoisomerase II enzyme—also known as DNA gyrase composed of two subunits encoded by *gyrA* and *gyrB*—leading to cellular death by forming double-strand DNA breaks [21]. Whereas beta-lactam antibiotics—including penicillins, cephalosporins, carbapenems, and monobactams—act by binding to and inhibiting the penicillin binding proteins (PBP) leading to stop in cross-linking or transpeptidations within the bacterial cell wall, and thus undergo cellular death [22]. Furthermore, bactericidal antibiotics interfere with common metabolic systems such as the central metabolic pathways called tri-carboxylic acid (TCA) cycle and iron metabolism. For example, reactive oxygen radicals in response to lethal bactericidal antibiotics result in cellular death [23,24].

Rifamycin is a semi-synthetic bactericidal class of antibiotic that can induce cell death by inhibiting bacterial RNA synthesis [65]. During the execution of the normal cellular function, beta-subunit of DNA-dependent RNA polymerase enzyme is involved in the stable channel formation between RNA-polymerase and DNA complex from which newly synthesized RNA strand arises [66,67]. Rifampicin stably binds to the beta-subunit of DNA dependent RNA polymerase (encoded by *rpoB* gene), thus inhibiting the high-fidelity transcription and causing cellular death.

Another class of bactericidal antibiotic such as aminoglycoside causes bacterial cellular death by interfering with cellular energetics, ribosome binding and protein synthesis inhibition [68]. Bacterial protein synthesis through the translation of mRNA occurs in a sequential fashion involving initiation, elongation, and termination. This process is operated in the cytoplasmic space involving the collaborative action of the ribosome (which acts as a factory) and many other important accessory translation factors available in the cytoplasm [69]. The ribosome is composed of two ribonucleoprotein subunits called the 30S (encoded by *rpsL* gene) and 50S. Following the formation of a complex between mRNA-transcript, N-formyl methionine-charged aminoacyl tRNA, several initiation factors and a free 30S subunit (this process is called initiation step of translation), the ribosome is assembled for the next translational step [70]. While translation is a complex process that requires many cellular components and translation factors, drugs can interfere with protein synthesis in various ways. Antibiotics that inhibit protein synthesis are classified into the 50S and 30S inhibitors, respectively. The 50S inhibitors (i.e., erythromycin, clindamycin, streptogramin, chloramphenicol, and linezolid) interfere with protein synthesis by blocking the initiation of protein translation or translocation of peptidyl tRNAs [71,72]. Inhibition of 30S ribosome (i.e., caused by tetracyclines and aminocyclitols) involves blocking of the access of aminoacyl tRNAs to the ribosome. Both spectinomycin and aminoglycosides—including streptomycin, kanamycin, and gentamycin—bind to the 16S rRNA, a component of the 30S ribosomal subunit. Specifically, aminoglycosides bind to the 16S rRNA, which in turn alter the conformation of the complex formed between an mRNA codon and its cognate charged aminoacyl tRNA in the ribosome. This interaction results in defective protein [19,26,73], thus cellular death occurs.

Polymyxins group of antibiotics (polymyxin A–E) are of cationic cyclic polypeptide origin with strong bactericidal effects. Among these, only polymyxin B and polymyxin E—also called colistin—are used in clinical practices [74]. Both antibiotics are commonly prescribed to treat infections caused by Gram-negative bacterial pathogens, particularly against extensively drug resistant (XDR) *P. aeruginosa* and *A. baumannii* [75]. These cyclic antimicrobial peptides have long hydrophobic tails which directly interact with LPS of the outer membrane of Gram-negative bacteria. Particularly, polymyxins destabilize calcium and magnesium bridge by binding to the lipid A component of the LPS. This event causes bacterial outer membrane permeabilization and allows polymyxins to enter the outer membrane, leading to cellular death [60].

**Figure 2:**
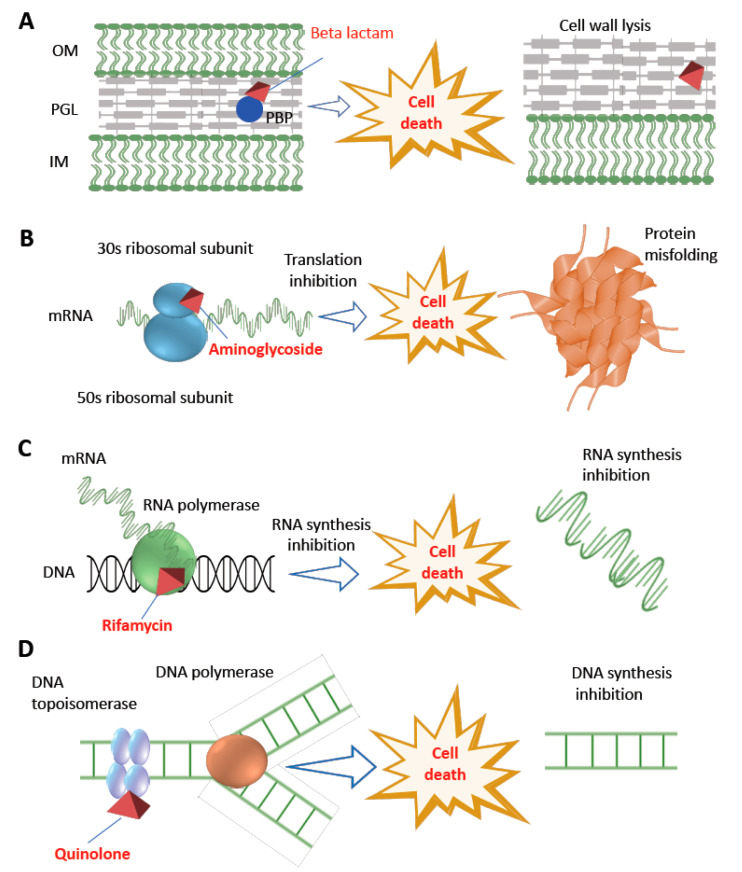
Diversity of antibiotic resistance mechanisms. The figure shows the major bactericidal antibiotics and their different targets. Beta-lactam antibiotics degrade bacterial cell wall by interfering with cross-linking or transpeptidations within the bacterial cell wall by binding with PBP (panel **A**), aminoglycoside interferes with protein synthesis by binding with 30S ribosomal subunit (panel **B**), rifamycin inhibits bacterial transcription by interfering with beta-subunit of DNA dependent RNA polymerase enzyme (panel **C**), whereas quinolone class of antibiotics inhibit DNA synthesis by interfering with DNA topoisomerase (panel **D**). OM: outer membrane; PGL: peptidoglycan layer; IM: inner membrane; PBP: penicillin binding protein. Mechanism of action of polymyxin and daptomycin is provided in the text.

Daptomycin is another class of bactericidal antibiotic and is composed of cyclic polypeptides. Daptomycin is used for the treatment of infections caused by Gram-positive bacteria, particularly against methicillin resistance *S. aureus* and *Enterococcus faecium*. Daptomycin interacts with anionic phospholipids in the presence of calcium ions in the cytoplasmic membrane. This interaction helps daptomycin penetrate the membrane, which ultimately causes membrane depolarization and cellular death. However, unlike most other bactericidal antibiotics, the detailed mechanistic basis of cellular death and resistance for daptomycin is not yet fully elucidated [76]. Detailed mechanisms of action of the major classes of bactericidal antibiotics are depicted in Figure 2.

## 3. Bacteria Employ Diverse Devices to Resist Antibiotics and Spread Antibiotic Resistance

Antibiotic resistance can be defined as the property inherent in bacteria by which successful uses of antibiotics are compromised by the development of tolerance or resistance against them [7,77]. Generally, antibiotic resistance is associated with prolonged exposure to antibiotics. More specifically, a bacterial population remains susceptible to antibiotics at the beginning of treatment but can sustain and develop resistance against these antibiotics gradually. Therefore, the continuous selective pressure exerted by antibiotics has helped bacteria develop resistance to one or more drugs simultaneously [78]. In recent years, the types and mechanisms of antibiotic resistance have become more complex due to the rapid emergence of new mechanisms in bacterial populations. Innate or intrinsic resistance and acquired resistance are two major types of antibacterial resistance mechanisms employed by bacteria to inhibit the lethal effects of antibiotics. Intrinsic resistance is a naturally occurring trait which refers to bacterial ability to withstand the lethal effects of antibiotics due to its structural or functional features. Therefore, intrinsic resistance is not related to previous antibiotic exposure or horizontal gene transfer. Gram-positive bacteria are susceptible to various antibiotics because of their inbuilt architecture of peptidoglycan, which offers large permeability of various antibiotics. In Gram-negative bacteria, the outer membrane (lipopolysaccharide, LPS) acts as a protective filter and provides intrinsic resistance to many antibiotics. Efflux pumps of Gram-negative bacteria also provide natural resistance to many antibiotics [79]. In the following sections, we will discuss more about intrinsic and acquired resistance to various antibiotics.

### 3.1. Natural Defense Systems to Antibiotics

**Figure 3:**
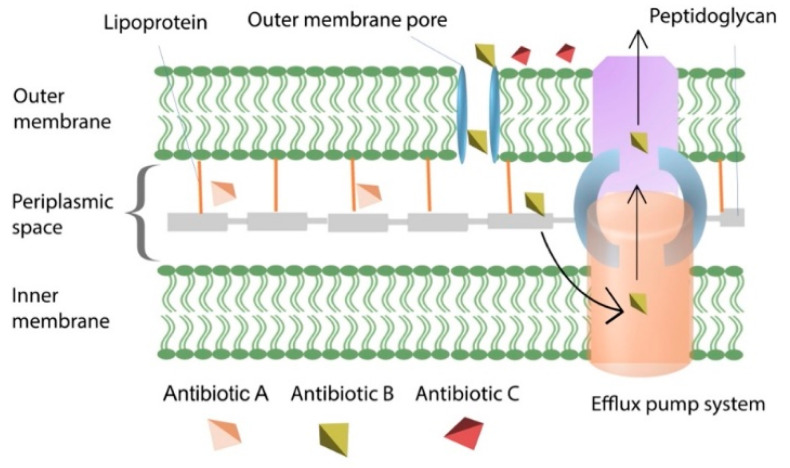
Active efflux pumping system to eliminate antibiotics from the periplasm. Efflux pump is associated with intrinsic antibiotic resistance. Intrinsic resistance is considered as phenotypic resistance as tolerance is not mediated by any genetic mutation. A = beta-lactam antibiotic which binds to the penicillin binding protein (PBP) and destabilizes peptidoglycan; B = aminoglycoside antibiotic; C = polymyxin antibiotic. Most notably, decreased susceptibility mediated by efflux system is mostly linked with aminoglycosides and fluoroquinolone, which is predominantly observed in Gram-negative bacteria.

Naturally occurring genes in the chromosome of the bacterial host confer intrinsic resistance. All bacterial species exhibit intrinsic resistance to specific antimicrobial classes. The biology of these mechanisms differs among bacteria. Some bacteria confer resistance by increased expression of efflux pump systems, which drive antibiotics out of the cells before they reach the target location, whereas others lack appropriate transport systems. As a result, antibiotics cannot penetrate the cell wall and reach the target spots. Several bacteria have genes or enzymes that confer innate resistance to antibiotics. Streptomyces, for example, has genes that confer resistance to streptomycin, which it produces. Beta-lactamase and inducible AmpC cephalosporinases, which hydrolyze beta-lactam antibiotics in the periplasmic space and are carried by Gram-negative bacteria, are other examples [80]. Another type of intrinsic resistance commonly observed in bacteria is impermeability. Impermeability occurs when antimicrobial chemicals are unable to enter through the bacterial outer membrane. For example, vancomycin can only target peptidoglycan crosslinking in Gram-positive bacteria by binding to the d-ala-d-ala peptide chain; however, vancomycin cannot penetrate through the outer membrane and reach peptides in the periplasm in Gram-negative bacteria [81]. Bacterial intrinsic mechanism of resistance via efflux pump system is a significant resistance mechanism, predominantly observed in Gram-negative bacteria. While an antibiotic can pass through a membrane-spanning porin protein called outer membrane protein (OMP) and reach the periplasmic area, it is eliminated or pumped out of the periplasm by many active efflux mechanisms. For example, intrinsic resistance to tetracycline, chloramphenicol and norfloxacin is well evident in Gram-negative *P. aeruginosa*, and this process is mediated by chromosomally encoded MexAB-OprM efflux system. Figure 3 portrays different examples of inherent resistance mechanisms. For example, a beta-lactam antibiotic (i.e., depicted as antibiotic A) that targets a penicillin-binding protein (PBP) channels through the outer membrane proteins (porins) and binds to the PBP target site, interfering with bacterial cell wall synthesis—a natural mechanism of action for beta-lactam antibiotics. Whereas antibiotic B (aminoglycoside antibiotic) can pass through the porin channel but is efficiently pumped out of the periplasm via the efflux pump. There are some antibiotics, such as polymyxins class (i.e., portrayed as antibiotic C) cannot cross the bacterial outer membrane [82].

### 3.2. Bacteria Acquire High Levels of Antibiotic Resistance through De Novo Mutations and Horizontal Gene Transfer

Bacteria can acquire resistance through spontaneous mutations, which result from errors in the cellular and metabolic processes such as DNA replication, transcription, recombination [83]. For instance, mutations in the target genes alter the binding site of antibiotics, or mutations at the target sites can lead to the overexpression of targets during the transcriptional step. These targets otherwise are naturally expressed at a very low level. In some instances, specific mutations can modify the drug targets. As a result, the minimum inhibitory concentration (MIC) of a particular antibiotic increases beyond the therapeutic limit [84]. In *Enterobacter* sp. and *P. aeruginosa*, overexpression of the *blaAmpC* is caused by mutation in the regulatory gene. In the presence of AmpC cephalosporinases, this overexpressed gene disrupts enzyme-to substrate ratio, consequently resistance to both penicillin and extended-spectrum cephalosporin occurs [85]. Other resistance mechanism such as mutation in penicillin binding protein 2b (*pbp2b*) gene can result in penicillin resistance in *Pneumococci*, whereas mutation in gene encoding ribosomal protein (i.e., *rpsL*) triggers altered conformational change for the drug-target binding interaction which in turn confers resistance to different aminoglycoside antibiotics. Altered efflux systems mediated by many different mutations can also cause up-regulation or overexpression of the efflux systems, therefore antibiotics cannot reach to the target sites of the bacterial cells [85]. Fluoroquinolone resistant *E. coli* and *Pseudomonas aeruginosa* are the two best examples of this type of resistance.

**Figure 4:**
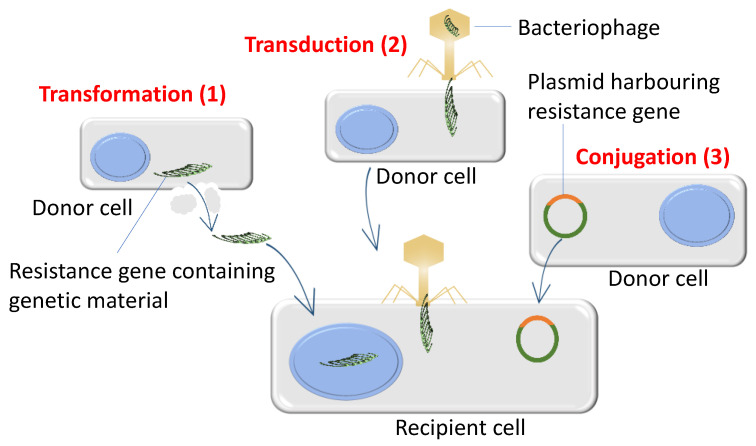
Trinity of horizontal resistance gene transfer modalities. Transmission of genetic material by horizontal genetic transfer, which is accomplished by three different mechanisms: transformation—bacteria take up naked DNA from the environment and integrate it to their chromosomes (1), transduction—bacteriophages carry resistance genes and transfer them to multiple hosts (2), and conjugation—resistance genes are transferred between bacterial cells through cell-to-cell contact (3).

**Figure 5:**
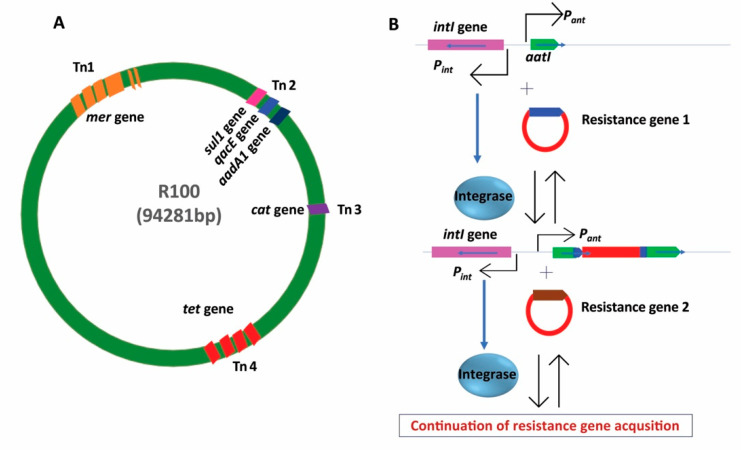
Antibiotic resistance via acquisition of mobile genetic elements (MGEs). The structure of a resistance plasmid (R100) (panel **A**) and the process of resistance gene acquisition (panel **B**) are illustrated. Resistant plasmid harbouring many different resistance genes as part of transposon (Tn) element can confer multidrug resistance by a single conjugation event. Integron mediated resistance gene capture system is frequently observed in many different clinical bacterial species. Integrase (transcribed under a downstream promoter (*Pint*)) catalyzes the insertion of an integron. Resistance gene cassette 1 (blue) is integrated into the *attI* site, which is under the influence of an upstream promoter (*Pant*). This way, many different resistance genes can be captured repeatedly for example, resistance gene 2. All resistance genes remain under the same promoter and thus become a resistance operon. Tn: transposon; bp: base-pair; *tet*: tetracycline resistance gene; *cat*: chloramphenicol acetyltransferase; *sul1*: sulphonamide resistance gene; *aadA1*: aminoglycoside adenylyltransferase; *mer*: mercury resistance gene; *qacE*: quaternary ammonium compound-resistance gene.

Bacteria can also develop resistance via the acquisition of resistance genes or resistance determinants externally. This process is known as horizontal gene transfer (HGT). HGT can occur through three main mechanisms: transduction, transformation and conjugation [86], as shown in Figure 4. Furthermore, new processes such as nanotubes [87] or extracellular vesicles mediated HGT have recently been reported [88]. During the transduction process, resistance gene transfer is mediated by bacteriophage which infects and transfers resistance genes to new bacterial species as witnessed in methicillin-resistant *Staphylococcus aureus* (MRSA) which developed resistance through the acquisition of resistance-conferring *mecA* gene from other bacterial species by transduction [89]. In another example, resistance genes from the dead bacterial DNA can be taken up and integrated into the recipient’s chromosomes by homologous recombination through a process called natural transformation. Acquired resistance by natural transformation is assumed to have frequently occurred in many clinical bacterial species. For example, *Streptococcus pneumoniae* is thought to have acquired penicillin-binding proteins (PBP2Bs) from the dead *Streptococcus mitis* [90], whereas the acquisition of the ceftriaxone resistance *penA* gene in *Neisseria gonorrhoeae* [91] was acquired through natural transformation. Lastly, bacteria acquire resistance genes or genetic determinants by conjugation which is mainly mediated by mobile genetic elements (MGEs) such as different types of plasmids and transposons [92,93]. The structure of MGEs is illustrated in Figure 5. To date, most of the resistance genes that disseminate to important clinical Gram-negative bacteria are of plasmid-borne conjugative resistance determinants origins. Among these, the notorious carbapenemase hydrolyzing enzymes in Gram-negative *Enterobacteriaceae* and in others Gram-negative bacteria have become the major global health threats because resistance genes harbouring plasmids can be rapidly disseminated to other susceptible bacteria through conjugation [94]. Particularly, conjugation-based plasmid-mediated antimicrobial resistance poses a serious threat to human health. Because enzymes (i.e., KPC carbapenemase and NDM-1 metallo-beta-lactamases) that hydrolyze carbapenems—the last resort antimicrobial class against Gram-negative bacteria—as well as other beta-lactams were found on different plasmids [95,96]. Transmission of resistance genes between plasmids and bacterial chromosomes via integrative chromosomal elements (ICEs) is also mediated by conjugation process (Figure 5). This mechanism of ICE-mediated resistance propagation mostly prevalent in Gram-negative bacteria, but in some instances, this can also be found in Gram-positive bacteria, as reported in *Streptococci* spp. [97,98].

## 4. Bacterial Resistance to Multiple Antibiotics via Diverse Biochemical Mechanisms

Simultaneous resistance to several antimicrobial compounds in diverse pathogenic bacterial populations has emerged as the major impediment for the successful treatment of infectious diseases globally. Since bacteria develop such resistance very rapidly by diverse resistance mechanisms, it has now become very difficult to treat even common infectious diseases [93]. More specifically, our current standard treatment protocols fail to produce a significant therapeutic response against those multidrug-resistant pathogenic bacteria. Treatment failure due to the evolution of multidrug-resistant bacteria also leads to a prolonged hospital stay with severe illness and can cause a higher risk of morbidity and mortality. The occurrence of resistance to multiple drugs has been reported in many clinical bacterial populations of both Gram-positive and Gram-negative origins. These populations have been defined as multidrug-resistant organisms (MDROs) when they show in vitro resistance to at least three or more antimicrobial classes, as described in the proceeding sections. The most dangerous MDR bacteria belong to the Gram-negative family, such as *P. aeruginosa*, *E. coli*, *A. baumannii*, and *K. pneumoniae*—a notorious producer of ESBLs, KPCs, and MBLs (i.e., NDM). In Gram-positive bacteria, methicillin resistant *S. aureus* (MRSA), vancomycin resistant *enterococci* (VRE), and extensively drug resistant *M. tuberculosis* (XDR) are notable examples of the MDR bacteria [85,99,100]. Resistance to multiple antibiotics (MDR) is mediated by a diverse array of mechanisms such as enzymatic mechanisms of drug modification, target modification by mutations, enhanced efflux-pump expression and altered membrane permeability [101].

### 4.1. Disabling Antibiotic Activities through Enzymatic Modification

Enzymatic modification of antibiotics has been reported in many clinical bacterial populations. Such resistance mechanisms have commonly but widely been documented for natural antibiotics, including aminoglycosides (i.e., kanamycin, amikacin, and tobramycin) and beta-lactam antibiotics. In each case, certain enzymes can modify the chemical composition of the antibiotics, which in turn result in altered drug-target interactions [102]. Commonly observed aminoglycosides modifying enzymes are aminoglycoside acetyltransferase (AAC-3-II), aminoglycoside phosphorylase (APH-3′-I) and adenylate (nucleotidyl transferases). For example, AAC-3-II can modify different aminoglycosides including amikacin, gentamycin, and tobramycin. This enzyme is mostly carried on MGEs and confer resistance to multiple antibiotics. It has been reported that multiple acetyltransferases encoding genes were harboured on class-1 integron in clinical *P. aeruginosa*, which conferred resistance to many other classes of antibiotics, including carbapenems and sulfonamides [103]. Enzymatic inactivation of beta-lactam antibiotics is also common in certain multidrug-resistant clinical bacteria. Genes located on plasmids mostly encode the beta-lactam degrading enzymes, but chromosomal genes can also encode such enzymes. Most clinically relevant beta-lactam hydrolyzing enzymes are beta-lactamase (first reported in *E. coli* against penicillin), TEM beta-lactamase conferring resistance to multiple drugs is commonly found in Gram-negative bacteria containing also multidrug resistant R plasmids, and CTX-M beta-lactamase which was originally derived from the mobilization of chromosomal *bla* genes from *Kluyvera* spp. through mobile genetic elements. All these enzymes belong to the ESBL (extended-spectrum beta-lactamase) class of enzymes [94,104,105].

### 4.2. Bypassing Antibiotic Interactions by Altering Target Sites

Bacteria develop antibiotic resistance as a result of mutations that make antibiotic target sites less accessible. Mutational resistance has been observed in many multidrug-resistant bacteria. For example, mutations in DNA gyrase (comprised of GyrA and GyrB) and topoisomerase IV (comprised of ParC/GrlA and ParE/GrlB) give clinically relevant resistance to fluoroquinolones [21,106]. Most of these mutations conferring resistance to fluoroquinolone are in a region termed quinolone resistance determining region (QRDR) of GyrA and ParC/GrlA. Interestingly, mutations in DNA gyrase occur in Gram-negative bacteria, whereas mutations in topoisomerase IV appear in Gram-positive bacteria [93]. Another prominent example of resistance mediated by mutational target alteration is resistance to rifampicin antibiotics. During tuberculosis infection, rifampicin in combination with other drugs—including isoniazid, pyrazinamide, ethambutol, or streptomycin—remains the first-line therapy [107,108]. A mutation in the *rpoB* gene, on the other hand, induces a conformational shift in the RNA polymerase beta-subunit, which prevents rifampicin from binding to its target. Mutations conferring resistance to aminoglycosides are also common in many multidrug-resistant bacteria. For example, mutational alteration in small ribosomal protein (S12) encoded by *rpsL* gene as well as mutation in *rrs* gene confers resistance to streptomycin [51], which has been documented in *M. tuberculosis* [109].

### 4.3. Efflux Pumps Reduce Intracellular Antibiotic Concentration

Efflux systems are the significant contributors of natural antibiotic resistance in Gram-negative microorganisms, which can effectively ship numerous antimicrobial agents out of the cell [102]. Overexpression of efflux systems has been observed to be associated with undeniable level protection from numerous clinically significant antibiotics. Efflux systems in bacteria are grouped into two significant classes based on their substrate specificity: (i) substrate explicit efflux system can just vehicle certain antibiotics (e.g., Tet efflux system can just pump tetracycline antibiotics out of the cell); (ii) substrate unspecific efflux framework can expel numerous antimicrobials—called MDR efflux systems. Chromosomal genes typically encode such MDR efflux systems, however genes encoding MDR efflux systems can also move onto plasmids, which can ultimately spread to different microbes. There have been large numbers of such MDR efflux systems only found in the MDR bacterial populaces, as mentioned in the preceding section.

### 4.4. Alteration of Membrane Permeability Prevents the Penetration of Antibiotics into Bacterial Cells

Gram-negative bacteria are covered by two-membrane cell envelop (i.e., outer membrane and inner membrane). The outer membrane is composed of the asymmetrical bilayer of LPS and phospholipids. Porin proteins and uptake channels embedded into this outer membrane bilayer regulate the entry of both hydrophobic and hydrophilic compounds into the cell. However, during exposure to antibiotics, bacteria alter this permeability barrier through modification of porin proteins. Porin proteins help bacteria to transport different compounds in and out of the cell (Figure 3). Porin proteins are associated with multidrug resistance. For example, OmpF and OprD are commonly occurring porin proteins found in *E. coli* and *P. aeruginosa*, respectively. Both are involved in unspecific entry and exit points for many different antibiotics and small chemical molecules. The membrane of *P. aeruginosa* is about 10 to 80 times less permeable compared to *E. coli*. This restricted membrane permeability provides built-in protections to *P. aeruginosa* against many different antibiotics compared to *E. coli*. Overall, porin proteins embedded in the outer membrane are involved in both acquired and adaptive resistance to multiple drugs. For example, imipenem and meropenem are passed through this entry, and mutations can cause reduced levels of OprD expression that confers resistance to these drugs. Furthermore, reduced porin expression in *Pseudomonas* spp. and *Acinetobacter* spp., results in resistance to newer drugs, including carbapenems and cephalosporins. Particularly, carbapenem mediated selective pressure favours the emergence of mutations both in the genes encoding porin channels as well as in the genes that regulate porin expression. Mutations in porin encoding genes in the global *K. pneumoniae* lineages also contribute to the emergence of carbapenem resistance. Furthermore, outer membrane porin protein such as Omp38 (or OpcP)—a homolog of *E. coli* OmpF—mediates multidrug resistance in *Burkholderia* spp. There have been numerous other bacteria where multidrug resistance through altered membrane permeability is present [43,110].

## 5. Evolution of Antibiotic Resistance Is a Result of Natural Selection

Resistance to antibiotics is one of the best demonstrations of bacterial adaptation by natural selection, a process in which a population adapts better to its environment over time. Antimicrobial compounds exert selection pressure and thus drive the evolution of resistance in bacterial populations. In the presence of antibiotic selection pressure, mutations occur randomly through mismatch errors during DNA replication and repair. This is how genetic variability—the raw material for the evolution of antibiotic resistance—occurs in bacterial populations. At any time, a small number of individuals in a large population carry de novo mutations or genes that are acquired by the HGT (more details of the HGT will be discussed in the later section) from the surrounding environments. In the presence of antibiotics, strains with resistance mutations are selected while others are eradicated. Since resistant strains have the advantage to survive, they continue growing and take over the population.

The rate of antibiotic resistance varies in types of antibiotics and bacterial species. Exposure to a certain class of antibiotics results in hypermutator or mutator resistant genotypes in the bacterial populations. This means that antibiotics exert an inhibitory effect on bacterial physiology, which in response selects for mutator or hypermutator genotypes. Several antibiotics belonging to different chemical classes can induce mutagenesis effect in bacteria [23]. For example, a well-sought ROS (reactive oxygen species) oxidative damage is induced by a sub-inhibitory concentration of a bactericidal antibiotic, as well documented in *E. coli* [111], and a general stress response is also induced by such antibiotics [112]. Particularly, aminoglycosides trigger translational-stress-induced mutagenesis, fluoroquinolones cause mutagenesis through DNA disruption, and beta-lactam classes of antibiotics can induce SOS response in bacteria [113,114,115]. SOS response produces mutator phenotypes by inducing error-prone DNA polymerase II, IV, and VI. A special case in point is cystic fibrosis patients where hypermutator population of *P. aeruginosa* are generated by alteration in the common genes which are involved in mismatch repair (MMR) system comprising *mutS*, *mutL*, *mutH*, and *uvrD*; in other case, mutator alleles can also result from the presence of antimutator genes, such as *mutT*, *mutY*, and *mutM* [116,117,118]. Another example is when *E. coli* were confronted in rifampicin environment, a substantial proportion of the *mutS*– populations fixed either one of the two transversion mutations, but the *mutS*+ strain was fixed with a wide array of mutations [119]. This finding suggests that antibiotic resistance evolves through genetic mutations in response to antibiotics and survival of the fittest mutants occurs when natural selection acts on these mutations [114]. Mutation rate varies from bacteria to bacteria due to the MMR system, as has been observed in *E. coli* and *S. enterica* [120].

## 6. Horizontal Gene Transfer and Bacterial Recombination Promotes Evolutionary Adaptation in Antibiotic Environments

HGT plays an important role in bacterial adaptation [121,122], particularly the evolution of multidrug resistance. This process can act across species barriers [121,122,123], provides genetic variation for natural selection acting on, and thus speeds up the adaptation [124,125]. Through a single HGT, multiple traits can be transferred and integrated into the recipient’s chromosomes, thus allow the hosts to expand their ecological niches, thrive in harsh environments, or increase virulence [126,127]. Previously, HGT was proposed to occur in only some bacteria. However, whole-genome sequencing in various species has confirmed the pervasiveness and the dominant role of this mechanism over spontaneous mutations in bacterial evolution [128,129]. It is estimated that at least 60% of prokaryotic genes have undergone HGT [130]. Many different resistant determinants borne on MGEs (i.e., plasmids, transposons, and integrons) are transferred and disseminated by HGT within and between species. Furthermore, a recent study showed that HGT can act as a directional force that allows neutral and mildly deleterious mutations conferring antibiotic resistance to maintain in the populations at a very low frequency even without strong selection [131]. At any given time, when selection pressure changes, strains with favorable genotypes will rapidly spread.

Among various mechanisms of HGT, the acquisition of multidrug resistance through recombination—i.e., natural transformation—could bring more evolutionary benefits to the bacterial populations [132,133,134]. First, recombination can integrate single or multiple DNA fragments into the recipient’s chromosomes [128,135]. This process increases genetic variation in the populations and thus potentiates the adaptation to further environmental changes [131]. Second, recombination can prevent the accumulation of deleterious mutations in populations during adaptation [136]. Since non-recombining populations reproduce through vertical gene transfer—i.e., mother to daughter—the next generation carry the same set of mutations as their mother. Mutations occur at any time and are often more deleterious than beneficial [137]. Over time offspring maintain more deleterious mutations leading to the deceleration of the adaptation. This process does not occur in recombining populations because recombination can bring beneficial mutations into one chromosome [136]. By shuffling available genetic variants in populations, recombination also prevents clonal interference [133]. Due to the crucial advantages to the adaptation, bacterial recombination is considered as equivalence to sexual production in Eukaryote [138]. Despite the benefits, recombination is less likely considered in studies of antibiotic resistance. Note that approximately 70% of the most dangerous human pathogens are naturally competent [139]. It has been suggested that clinical *A. baumannii* acquired resistance to multiple drugs via transformation. Particularly, this bacterium acquired 45 different resistance conferring genes from other species including *E. coli*, *Pseudomonas* spp., and *Salmonella* sp. by transformation [140]. However, the dynamic of transferred genetic variants in these recombining populations has not been mentioned.

## 7. Factors Affecting Evolutionary Dynamics of Resistance in Bacteria

Mutation supply rate determines the genetic variability in the infecting clonal populations under antibiotic selective pressure, as mentioned earlier. Mutation supply rate is determined by population size and rates of mutation and HGT in bacteria. However, adaptive evolution of drug resistance, for example, the rate at which antibiotic resistance will evolve and spread in the populations, is determined by several other factors, including relative fitness of the resistant genotypes as the function of drug concentration, the strength of selection pressure, clonal interference, population bottleneck or transmission bottleneck, compensatory mutation, presence of epistasis, drug–drug interaction and coselection [141]. Relative fitness in the absence or presence of drugs is a key component in determining the emergence and spread of resistant populations at a given mutation supply rate [78]. When selective pressure is altered, the frequency of resistant population or reversibility is determined by the relative fitness cost. Consequently, fitness has been a key component in determining and predicting the evolutionary adaptation of pathogen populations of both laboratory and clinical origin [142,143,144,145,146].

Both natural and clinical bacterial populations face a wide array of selective pressures in their surroundings, for example, in the environment (water and soil) or in certain body compartments of humans or animals. Such concentration zones present in the environment and in different niches of the body compartments favour the evolution and amplification of drug resistant mutant subpopulations by reducing drug susceptibility in the populations. According to the pharmacodynamic model, the selective zone for the resistant subpopulations is called mutation selection window (MSW) [147]. The lower boundary of this MSW promotes the elimination of the susceptible bacteria, which is often referred to as minimum inhibitory concentration (MIC). According to traditional MSW (i.e., drug concentration ranges between the MIC of susceptible and the MIC of resistant bacterial populations—called mutant prevention concentration or the MPC), at high drug concentration, the rate of resistance emergence is determined by the pre-existing mutation in the populations. However, at low selective pressure, populations are enriched with many small effect resistance mutations [148,149]. Consequently, it complicates the prediction of mutational paths of antibiotic resistance due to the diversity of resistant mutants [150]. Low level of drug concentration, which is typically many folds below the MIC of the susceptible bacteria, selects for resistance in the populations is called minimal selective concentration (MSC). Therefore, sub-MIC selective window is important for the evolution and maintenance of resistance in bacterial populations [151]. Furthermore, it has been evident that strong selection pressure favours high level of cross-resistance (this can also be termed as negative collateral sensitivity) to many antimicrobial classes, whereas under low selection pressure populations enrich with weaker cross-resistance [152]. Together, this suggests that the emergence and spread of resistant population is attributed to the strength of selection pressure encompassing both the sub-MIC selective window and the traditional MSW. Resistance to multiple drugs is the result of the concomitant presence of multiple resistance conferring mutations in the individual clonal lineage of a given population [153]. When different beneficial mutations (i.e., a mutation is beneficial when it appears in an individual bacterium in response to an antibiotic) arise in the populations, they compete against each other, leading to the loss of most clones from the population. However, clones with higher fitness outcompete the appearance of other clones with smaller fitness effect [154]. This phenomenon is called clonal interference and has been well sought in experimental resistant P. aeruginosa when evolved in the absence of antibiotic [155,156].

Epistasis—where the fitness effect of a mutation is influenced by another mutation present in other genetic location—plays an important role in the evolution of antibiotic resistance, multidrug resistance in particular [157]. Epistasis can occur between genes [158], within a single gene encoding a single resistance protein [159,160], or between a chromosomal gene and a gene encoded on a plasmid [161]. Several studies have identified pervasive epistasis in bacterial adaptive evolution under a variety of conditions. For example, in two studies, positive epistasis (when a double mutant has higher fitness than expected from the sum of the costs of individual mutations in the absence of selection pressure) was reported to the evolution of multidrug resistance [162,163]. Interestingly, this form of epistasis was observed between a resistance mutation and a tolerance mutation in the presence of antibiotic selection pressure, and the authors have termed this as synergistic interaction [164]. In another study, Marta Lukačišinová and colleagues used a high throughput platform to measure the evolvability of antibiotic resistance of hundreds of *E. coli* mutants from Keio collection in a high control environment of antibiotic concentration [165]. This study not only found the strong epitasis between resistance mutations but also identified genes that control the evolution of antibiotic resistance through spontaneous mutations. Importantly, strains that are more sensitive to antibiotics have a greater increase in resistance levels. This phenomenon is also known as diminishing-returns epistasis: the effects of beneficial mutations are stronger in less fit genetic backgrounds [166,167]. Although reduced use or withdrawing of antibiotic use has been suggested to reverse antibiotic resistance [78], epistasis plays a major role in determining the adaptive potential of resistant populations. For example, in some form of epistasis—called reciprocal sign epistasis—the fitness of multidrug resistant genotypes in the absence of drugs is greater than either of the singly resistant genotypes. This means that the acquisition of additional new resistance determinants (new resistance mutation or new resistance plasmid) can further accelerate the fitness of the initial resistant genotype. Therapeutic options become narrower when this form of epistasis arises in clinical bacterial pathogens.

Compensatory evolution is another important means of adaptive evolution of antibiotic resistance which also involves epistasis. Antibiotic resistance is deleterious (on bacterial fitness) in the absence of drug pressure. For example, in the absence of drug pressure, resistance determinants often impose fitness costs in the form of reduced growth, transmission, or virulence [168]. However, after certain generations intra or inter genomic secondary mutations arise in the resistant bacterial population that restore the costs incurred by the initial drug specific resistance mutations. This phenomenon of adaptive evolution has been reported in both in vivo and in vitro studies [142,169,170,171]. Compensatory mutations can be resistance mutations themselves, which can both compensate and confer resistance to other antibiotics. For example, in the absence of antibiotic A, resistance mutation incurs fitness cost, but after certain generations, this cost is compensated for by a new secondary mutation. This secondary mutation simultaneously ameliorates the fitness cost as well as confers resistance to a new antibiotic (i.e., antibiotic B). This form of pleiotropic fitness effect of a compensatory mutation has recently been observed between a streptomycin resistance mutation and a rifampicin resistance mutation in *E. coli* [163]. Moreover, it has been shown that compensatory mutations in multidrug resistance are distinct from the single drug [172]. Therefore, restricted utilization of antibiotics might not be an advocated solution to the antibiotic resistance crisis when compensatory mutations have already evolved in the dominant resistant strains [86].

Drug interactions are important in determining bacterial evolutionary adaptation to multiple drugs. Drug interactions have been classified into two types: physiological interactions and evolutionary interactions [173]. During physiological interaction, two antibiotics are used in combination, and they can produce either synergistic interaction, antagonistic interaction, or they can suppress each other’s effect—called suppressive drug interaction. Antibiotic drug interactions result from when the combined inhibitory effect of two drugs is larger (called synergistic interaction which is more inhibitory) or smaller (called antagonistic interaction, where higher MIC is needed to obtain the same level of inhibition of synergistic drug pair) than expected based on an additive model. During suppressive drug interaction, two drugs in combination produce a weaker effect, which is less than the null additive expectation or weaker than the individual drug effect. It has been reported that synergistic drug pairs, at a certain concentration threshold, potentiate the evolution of resistance by extending the traditional mutant selection window towards the sub-inhibitory concentration [174,175]. These studies have shown that during combination therapy (i.e., drug A + drug B) certain drug specific resistance mutations arise first (i.e., resistance mutation evolves to drug A), which in turn diminishes the synergistic action of that drug pair owing to the evolution of a drug specific resistance mutation at the first place. Subsequently, this mutation confers an enhanced growth advantage against that drug pair, which drives the acquisition of resistance mutation against the second drug B, thus multiple resistance mutations evolve in the presence of a synergistic drug pair. Synergistic drug pairs when used at low concentrations (i.e., below the MIC) can accelerate resistance emergence. On the contrary, it has been suggested that with antagonistic drug pair, certain mutations or mutations to drug A occur first which eventually breaks and convert antagonistic interactions into synergistic. Thus, antagonistic drug pairs decelerate resistance evolution to the second drug B. Evolutionary interactions are further classified into two types: cross-resistance and collateral sensitivity [173]. Resistance mutations or genes arising through either spontaneous mutation or HGT can simultaneously confer resistance to another drugs (called cross-resistance) or become more sensitive to other drugs (called ‘collaterally sensitive’) [176,177]. Cross-resistance is the function of the evolutionary response to a single antibiotic. Therefore, cross-resistance is different from the physiological interactions, which require drugs to be administered in combination. Cross-resistance jeopardizes the efficacy of antibiotic treatments, while collateral sensitivity provides the chance to prolong the effects of existing antibiotics as well as slow down the rate of multidrug resistance [178,179]. Collateral sensitivity has been found to be pervasive, however, its mechanisms and applications are not well understood [180]. For instance, the evolutionary trade-off of resistance to ciprofloxacin *P. aeruginosa* is the increase in sensitivity to two other drugs, piperacillin and tobramycin [181]. Another example is a long-term experimental evolution in 24 days of *E. coli* in a morbidostat that automatically controls the antibiotic treatments [182]. In this study, multidrug resistance was found to be prevented by the trade-off of polymyxin B resistance. In both studies, the collateral sensitivity was found to be unidirectional only. A more recent study provides additional details into the factors that contribute to stabilizing the trade-off [183]. Importantly, the maintenance of original pressure incurred by the first drug can maximize the hypersensitivity to the second drug. Moreover, drug order is one of the important factors that can enhance or prevent multidrug resistance.

Evolutionary dynamics of antibiotic resistance further rely on factors such as coselection and bottleneck. Coselection is a phenomenon where one resistance conferring subunit (i.e., a particular resistance gene) is transferred with another subunit or another gene (this process is often called hitchhiking), because the former gene (i.e., resistance conferring gene) and the latter gene form the whole unit. Coselection is an important factor in maintaining the resistance gene in bacterial population. For example, under given drug pressure, a particular population will select for a resistance conferring mutation in a gene by direct selection, or this gene will be selected with other genes (i.e., selection acts on a gene that is co-linked with other genes). Coselection occurs within the genes localized on a chromosome or between chromosomal genes and genes harboured on a plasmid, which are closely associated with each other. A resistance conferring gene always incurs a fitness cost, and coselection shields this resistance gene from purifying selection [184]. When sequential drug perturbation occurs, coselection favours populations to select resistance for different drugs in the co-linked genes. Thus, coselection favours the evolutionary maintenance of genes conferring resistance to multiple drugs.

Population bottleneck is another important determinant for the adaptive evolution of bacteria. For several reasons, this event is frequently observed in pathogenic bacteria, including transmission from one host to another host, immune pressure, and antibiotics. In the presence of antibiotic selective pressure, only resistant variants with higher fitness will flourish in the population. However, the probability of the occurrence of this resistance mutation in the subsequent generations is dependent on the population size or population bottleneck. In the presence of a narrow transmission bottleneck, populations with higher mutation rates (i.e., most commonly occurring mutants but they have low fitness) can pass onto the next generation. With a wider transmission bottleneck, both commonly occurring mutants with low fitness and rare mutants with higher fitness can transmit. In this scenario, the fittest genotypes will increase their frequency and will subsequently displace the genotypes with lower fitness. This competitive exclusion of low-fitness genotypes can be minimized through the HGT and recombination. Depending on the size, the population bottleneck also influences the occurrence of antibiotic resistance evolution in both laboratory and clinical bacterial populations. Overall, examples of such event can be drawn from several studies, for example multistep fluoroquinolone resistance in *E. coli* [143,185] and rifampicin resistance in *P. fluorescence* [186].

## 8. Future Landscapes

Ever-increasing antibiotic resistance cases and a slowdown pipeline of novel antibiotics have resulted in a shrinking toolkit to counter key clinically resistant bacteria. Thus, it is crucial to elucidate the evolutionary mechanisms of origin, risk concerns and continued supervision of antibiotic resistance to prolong the effective life of currently prevailing antibiotics. Additionally, numerous bacterial species upon selection for antibiotic resistance have hefty population density, shorter reproductive cycle, and robust selective pressures, rendering them reasonable models for exploring more extensive, fundamental inquiries with respect to fast adaptation against new selection pressures. This might incorporate inquiries about the significance of de novo mutation and standing variations, the causes underlying their variable prevalence in separate occasions, which adaptive choices are available to an emerging bacterial population, etc. Identifying the probable sources of antibiotic resistance before a clinical approval is adopted would expand the chances of fruitful proactive antibiotic resistance management. If we can predict the evolutionary trends of resistance of an antibiotic in bacterial species, it will be easy to customize antibiotic dosing regimens and to achieve an extended duration of use. Statistical modelling of medico-epidemiological data can generate a decent understanding of the relationship between the rate of resistance evolution and geographical spread pattern. The rate of resistance evolution in developing countries can be different from the developed countries because the antibiotic consumption rate is much higher in developing countries viz. Brazil, India, and China. Further research is required to understand the extent of concerns and repercussions to cope with the threats of resistance. This review will provide a distinctive reference for advancing healthcare settings, determining treatment regimes, and achieving a clearer insight into the fluctuating epidemiology of resistant bacteria.

## Figures and Tables

**Table 1:** Action and common resistance mechanisms of major bacteriostatic antibiotics.

Bacteriostatic Candidates	Mode of Action	Mechanism of Resistance
Tetracycline	Reversibly inhibits 30S ribosomal subunit of bacteria [25].	Efflux system and protecting ribosomes [26].
Macrolides	Reversibly inhibits 50S ribosomal subunit of bacteria [27].	Methylation of the 23S rRNA, efflux system [28].
Sulphonamides	Inhibits folate synthesizing enzyme dihydropteroate synthase (DHPS) [29].	By horizontal transfer of dihydropteroate synthase gene [30].
Streptogramins	Reversibly inhibits 50S ribosomal subunit of bacteria [31].	Acetyltransferases *vatD* gene expression mediates streptogramin A, wheras *vatE* and *ermB* or *vgbA* gene cluster confers streptogramin B antibiotics [32].
Oxazolidinones	Reversibly inhibits 50S ribosomal subunit of bacteria [33].	High diversity and coselection of *optrA* [34].
Lincosamides	Reversibly inhibits 50S ribosomal subunit of bacteria [35].	Target site modification, efflux system and drug inactivation [36].
Trimethoprim	Occupying the active site of bacterial dihydrofolate reductase (DHFR), thus blocking the activity of the enzyme [37].	Increase expression of DHFR or decrease the affinity of DHFR to the drug [38].

**Table 2:** Action and common resistance mechanisms of major bactericidal antibiotics.

Bactericide Candidates	Mode of Action	Mechanism of Resistance
Penicillins	Competitively inhibits the transpeptidase enzyme resulting cross-linking blockage in cell wall [39].	Beta-lactamase encoded by *blaZ*, altered PBP2a encoded by *mecA* [14,40], extended-spectrum-beta-lactamases (ESBLs), AmpC beta-lactamase (i.e., *blaAmpC*) [41,42,43].
Cephalosporins	Competitively inhibits the transpeptidase enzyme resulting in cross-linking or blockage in cell wall [44].	AmpC beta-lactamase (i.e., *blaAmpC*), ESBLs (i.e., *blaCTX-M*) [41,42].
Carbapenems	Binding with penicillin-binding proteins (PBPs) and inactivation of these proteins leads to cell wall synthesis interruption [45].	Carbapenemases (i.e., class A serine-carbapenemase including KPCs; class B metallo-carbapenemase including New-Delhi-metallo-beta-lactamases or NDM, Verona-integron-encoded beta-lactamases or VIM, Imepenemase IMP-carbapenemase (also a metallo-beta-lactamase); class D serine carbapenemase such oxacillinase (OXA) [46,47], mutation-derived target enzyme modification [48]; preventing the drug entry by modifying outer membrane permeability [49]; pumping carbapenems out by efflux pump systems [50].
Aminoglycoside	Binding with 30 s ribosomal subunit resulting genetic code misreading followed by interruption of bacterial translation [51].	Mostly through aminoglycosides modifying enzymes encoded by *aac* (aminoacetyl-tranferase) and *aph* (aminophospho-transferase), efflux system, or mutation in *rpsL* and 16S rRNA [43,52].
Fluoroquinolones	Interrupting bacterial DNA replication by inhibiting topoisomerases [53].	Target enzyme mutation (DNA gyrase encoded by *gyrA* and *gyrB*, and topoisomerase IV encoded by *parC* and *parE* genes), efflux system and changing drug entry [54].
Rifamycin	Interrupting transcription by inhibiting bacterial RNA polymerase [55].	Mutation of the target (beta subunit of RNA polymerase encoded by *rpoB*) [56].
Polymyxins	Binding to lipid A of LPS and interfere with outer membrane permeability [57].	The *pmrHFIJKLM* (also known as *arn* operon) and *pmrCAB* operon—both invove in the biosynthesis of LAra4N and modify the lipid A, thus disrupt lipid A charges [17]; mutations in genes encoding the two-component regulatory systems such as *pmrAB* [58], *phoPQ* and plasmid-borne *mcr* genes confer resistance to colistin—the last line of drug [59,60].
Daptomycin	Binding to anionic phospholipids in the cytoplasmic membrane [61].	Mutations in gene *mprF* which encodes the multiple peptide resistance factor [62].
Vancomycin	Binding to the dipeptide terminus d-Ala-d-Ala of peptidoglycan pentapeptide precursors preventing peptidoglycan crosslinking leads to the inhibition of bacterial cell wall synthesis [63].	Replacing d-Ala-d-Ala with d-Ala-d-lac or d-Ala-d-Ser alternatives to which vancomycin has low affinity [64].

## References

[1] Katz L., Baltz R.H. (2016). Natural product discovery: Past, present, and future. J. Ind. Microbiol. Biotechnol..

[2] Boucher H.W., Talbot G.H., Bradley J.S., Edwards J.E., Gilbert D., Rice L.B., Scheld M., Spellberg B., Bartlett J. (2009). Bad bugs, no drugs: No ESKAPE! An update from the Infectious Diseases Society of America. Clin. Infect. Dis..

[3] Lesho E.P., Laguio-Vila M. (2019). The Slow-Motion Catastrophe of Antimicrobial Resistance and Practical Interventions for All Prescribers. Mayo Clin. Proc..

[4] Giske C.G., Monnet D.L., Cars O., Carmeli Y. (2008). Clinical and economic impact of common multidrug-resistant gram-negative bacilli. Antimicrob. Agents Chemother..

[5] Årdal C., Balasegaram M., Laxminarayan R., McAdams D., Outterson K., Rex J.H., Sumpradit N. (2020). Antibiotic development—Economic, regulatory and societal challenges. Nat. Rev. Microbiol..

[6] O’Neill J. (2016). Tackling Drug-Resistant Infections Globally: Final Report and Recommendations: The Review on Antimicrobial Resistance.

[7] Kohanski M.A., Dwyer D.J., Collins J.J. (2010). How antibiotics kill bacteria: From targets to networks. Nat. Rev. Microbiol..

[8] Lawrie R. (1985). First clinical use of penicillin. Br. Med. J..

[9] Hutchings M.I., Truman A.W., Wilkinson B. (2019). Antibiotics: Past, present and future. Curr. Opin. Microbiol..

[10] Levy S.B. (2001). Antibiotic Resistance: Consequences of Inaction. Clin. Infect. Dis..

[11] Ventola C.L. (2015). The antibiotic resistance crisis: Part 1: Causes and threats. Pharm. Ther..

[12] Kwon J.H., Powderly W.G. (2021). The post-antibiotic era is here. Science.

[13] Abraham E.P., Chain E. (1940). An Enzyme from Bacteria able to Destroy Penicillin. Nature.

[14] Lobanovska M., Pilla G. (2017). Penicillin’s discovery and antibiotic resistance: Lessons for the future?. Yale J. Biol. Med..

[15] Rammelkamp C.H., Maxon T. (1942). Resistance of Staphylococcus aureus to the Action of Penicillin. Proc. Soc. Exp. Biol. Med..

[16] Biswas S., Brunel J.M., Dubus J.C., Reynaud-Gaubert M., Rolain J.M. (2012). Colistin: An update on the antibiotic of the 21st century. Expert Rev. Anti. Infect. Ther..

[17] Falagas M.E., Rafailidis P.I., Matthaiou D.K. (2010). Resistance to polymyxins: Mechanisms, frequency and treatment options. Drug Resist. Updates.

[18] Baquero F., Levin B.R. (2021). Proximate and ultimate causes of the bactericidal action of antibiotics. Nat. Rev. Microbiol..

[19] Davis B.D. (1987). Mechanism of bactericidal action of aminoglycosides. Microbiol. Rev..

[20] Walsh C. (2003). Antibiotics.

[21] Drlica K., Malik M. (2003). Fluoroquinolones: Action and Resistance. Curr. Top. Med. Chem..

[22] Tomasz A. (1979). The mechanism of the irreversible antimicrobial effects of penicillins: How the beta-lactam antibiotics kill and lyse bacteria. Annu. Rev. Microbiol..

[23] Kohanski M.A., Dwyer D.J., Hayete B., Lawrence C.A., Collins J.J. (2007). A Common Mechanism of Cellular Death Induced by Bactericidal Antibiotics. Cell.

[24] Kohanski M.A., Dwyer D.J., Wierzbowski J., Cottarel G., Collins J.J. (2008). Mistranslation of Membrane Proteins and Two-Component System Activation Trigger Antibiotic-Mediated Cell Death. Cell.

[25] Smilack J.D. (1999). The Tetracyclines. Mayo Clin. Proc..

[26] Chopra I., Roberts M. (2001). Tetracycline Antibiotics: Mode of Action, Applications, Molecular Biology, and Epidemiology of Bacterial Resistance. Microbiol. Mol. Biol. Rev..

[27] Smieja M. (1998). Current indications for the use of clindamycin: A critical review. Can. J. Infect. Dis..

[28] Fyfe C., Grossman T.H., Kerstein K., Sutcliffe J. (2016). Resistance to macrolide antibiotics in public health pathogens. Cold Spring Harb. Perspect. Med..

[29] Brock Madigan M.T., Martinko J.M., Stahl D.A., Clark D.P. (2009). Brock Biology of Microorganisms.

[30] Sköld O. (2000). Sulfonamide resistance: Mechanisms and trends. Drug Resist. Update.

[31] Vannuffel P., Cocito C. (1996). Mechanism of action of streptogramins and macrolides. Drugs.

[32] Werner G., Klare I., Witte W. (2002). Molecular analysis of streptogramin resistance in enterococci. Int. J. Med. Microbiol..

[33] Pandit N., Singla R.K., Shrivastava B. (2012). Current Updates on Oxazolidinone and Its Significance. Int. J. Med. Chem..

[34] Chen H., Wang X., Yin Y., Li S., Zhang Y., Wang Q., Wang H. (2019). Molecular characteristics of oxazolidinone resistance in enterococci from a multicenter study in China. BMC Microbiol..

[35] Schlünzen F., Zarivach R., Harms J., Bashan A., Tocilj A., Albrecht R., Yonath A., Franceschi F. (2001). Structural basis for the interaction of antibiotics with the peptidyl transferase centre in eubacteria. Nature.

[36] Leclercq R. (2002). Mechanisms of resistance to macrolides and lincosamides: Nature of the resistance elements and their clinical implications. Clin. Infect. Dis..

[37] Manna M.S., Tamer Y.T., Gaszek I., Poulides N., Ahmed A., Wang X., Toprak F.C.R., Woodard D.R., Koh A.Y., Williams N.S. (2021). A trimethoprim derivative impedes antibiotic resistance evolution. Nat. Commun..

[38] Tamer Y.T., Gaszek I.K., Abdizadeh H., Batur T.A., Reynolds K.A., Atilgan A.R., Atilgan C., Toprak E. (2019). High-Order Epistasis in Catalytic Power of Dihydrofolate Reductase Gives Rise to a Rugged Fitness Landscape in the Presence of Trimethoprim Selection. Mol. Biol. Evol..

[39] Yocum R.R., Rasmussen J.R., Strominger J.L. (1980). The mechanism of action of penicillin. Penicillin acylates the active site of Bacillus stearothermophilus D-alanine carboxypeptidase. J. Biol. Chem..

[40] Lowy F.D. (2003). Antimicrobial resistance: The example of Staphylococcus aureus. J. Clin. Investig..

[41] Jacoby G.A. (2009). AmpC β-Lactamases. Clin. Microbiol. Rev..

[42] Canton R., Gonzalez-Alba J.M., Galán J.C. (2012). CTX-M Enzymes: Origin and Diffusion. Front. Microbiol..

[43] Kakoullis L., Papachristodoulou E., Chra P., Panos G. (2021). Mechanisms of Antibiotic Resistance in Important Gram-Positive and Gram-Negative Pathogens and Novel Antibiotic Solutions. Antibiotics.

[44] Yotsuji A., Mitsuyama J., Hori R., Yasuda T., Saikawa I., Inoue M., Mitsuhashi S. (1988). Mechanism of action of cephalosporins and resistance caused by decreased affinity for penicillin-binding proteins in Bacteroides fragilis. Antimicrob. Agents Chemother..

[45] Papp-Wallace K.M., Endimiani A., Taracila M.A., Bonomo R.A. (2011). Carbapenems: Past, Present, and Future. Antimicrob. Agents Chemother..

[46] Nordmann P., Dortet L., Poirel L. (2012). Carbapenem resistance in Enterobacteriaceae: Here is the storm!. Trends Mol. Med..

[47] Marie Q.A., Karen B. (2007). Carbapenemases: The Versatile β-Lactamases. Clin. Microbiol. Rev..

[48] Meletis G. (2015). Carbapenem resistance: Overview of the problem and future perspectives. Ther. Adv. Infect. Dis..

[49] Bonomo R.A., Szabo D. (2006). Mechanisms of Multidrug Resistance in Acinetobacter Species and Pseudomonas aeruginosa. Clin. Infect. Dis..

[50] Walsh T.R. (2010). Emerging carbapenemases: A global perspective. Int. J. Antimicrob. Agents.

[51] Krause K.M., Serio A.W., Kane T.R., Connolly L.E. (2016). Aminoglycosides: An overview. Cold Spring Harb. Perspect. Med..

[52] Garneau-Tsodikova S., Labby K.J. (2016). Mechanisms of resistance to aminoglycoside antibiotics: Overview and perspectives. Medchemcomm.

[53] Aldred K.J., Kerns R.J., Osheroff N. (2014). Mechanism of Quinolone Action and Resistance. Biochemistry.

[54] Jacoby G.A. (2005). Mechanisms of Resistance to Quinolones. Clin. Infect. Dis..

[55] Wehrli W. (1983). Rifampin: Mechanisms of Action and Resistance. Rev. Infect. Dis..

[56] Tupin A., Gualtieri M., Roquet-Banères F., Morichaud Z., Brodolin K., Leonetti J.-P. (2010). Resistance to rifampicin: At the crossroads between ecological, genomic and medical concerns. Int. J. Antimicrob. Agents.

[57] Velkov T., Thompson P.E., Nation R.L., Li J. (2010). Structure-activity relationships of polymyxin antibiotics. J. Med. Chem..

[58] Bingbing S., Haiyan L., Yu J., Lei S., Sheng Y., Daijie C., Mariana C. (2021). New Mutations Involved in Colistin Resistance in Acinetobacter baumannii. mSphere.

[59] Andrade F.F., Silva D., Rodrigues A., Pina-Vaz C. (2020). Colistin update on its mechanism of action and resistance, present and future challenges. Microorganisms.

[60] Laurent P., Aurélie J., Patrice N. (2017). Polymyxins: Antibacterial Activity, Susceptibility Testing, and Resistance Mechanisms Encoded by Plasmids or Chromosomes. Clin. Microbiol. Rev..

[61] Randall C.P., Mariner K.R., Chopra I., O’Neill A.J. (2013). The Target of Daptomycin Is Absent from Escherichia coli and Other Gram-Negative Pathogens. Antimicrob. Agents Chemother..

[62] Mishra N.N., Yang S.-J., Chen L., Muller C., Saleh-Mghir A., Kuhn S., Peschel A., Yeaman M.R., Nast C.C., Kreiswirth B.N. (2013). Emergence of Daptomycin Resistance in Daptomycin-Naïve Rabbits with Methicillin-Resistant Staphylococcus aureus Prosthetic Joint Infection Is Associated with Resistance to Host Defense Cationic Peptides and mprF Polymorphisms. PLoS ONE.

[63] Blaskovich M.A.T., Hansford K.A., Butler M.S., Jia Z., Mark A.E., Cooper M.A. (2018). Developments in Glycopeptide Antibiotics. ACS Infect. Dis..

[64] Stogios P.J., Savchenko A. (2020). Molecular mechanisms of vancomycin resistance. Protein Sci..

[65] Floss H.G., Yu T.W. (2005). Rifamycin—Mode of action, resistance, and biosynthesis. Chem. Rev..

[66] Campbell E.A., Korzheva N., Mustaev A., Murakami K., Nair S., Goldfarb A., Darst S.A. (2001). Structural mechanism for rifampicin inhibition of bacterial RNA polymerase. Cell.

[67] McClure W.R., Cech C.L. (1978). On the mechanism of rifampicin inhibition of RNA synthesis. J. Biol. Chem..

[68] Vakulenko S.B., Mobashery S. (2003). Versatility of aminoglycosides and prospects for their future. Clin. Microbiol. Rev..

[69] Dunkle J.A., Xiong L., Mankin A.S., Cate J.H.D. (2010). Structures of the Escherichia coli ribosome with antibiotics bound near the peptidyl transferase center explain spectra of drug action. Proc. Natl. Acad. Sci. USA.

[70] Nissen P., Hansen J., Ban N., Moore P.B., Steitz T.A. (2000). The Structural Basis of Ribosome Activity in Peptide Bond Synthesis. Science.

[71] Menninger J.R., Otto D.P. (1982). Erythromycin, carbomycin, and spiramycin inhibit protein synthesis by stimulating the dissocation of peptidyl-tRNA from ribosomes. Antimicrob. Agents Chemother..

[72] Patel U., Yan Y.P., Hobbs F.W., Kaczmarczyk J., Slee A.M., Pompliano D.L., Kurilla M.G., Bobkova E.V. (2001). Oxazolidinones Mechanism of Action: Inhibition of the First Peptide Bond Formation. J. Biol. Chem..

[73] Karimi R., Ehrenberg M. (1994). Dissociation Rate of Cognate Peptidyl-tRNA from the A-Site of Hyper-Accurate and Error-Prone Ribosomes. Eur. J. Biochem..

[74] Falagas M.E., Kasiakou S.K. (2005). Colistin: The revival of polymyxins for the management of multidrug-resistant gram-negative bacterial infections. Clin. Infect. Dis..

[75] Gurjar M. (2015). Colistin for lung infection: An update. J. Intensive Care.

[76] Ernst C.M., Peschel A. (2019). MprF-mediated daptomycin resistance. Int. J. Med. Microbiol..

[77] Davies J., Davies D. (2010). Origins and evolution of antibiotic resistance. Microbiol. Mol. Biol. Rev..

[78] Andersson D.I., Hughes D. (2010). Antibiotic resistance and its cost: Is it possible to reverse resistance?. Nat. Rev. Microbiol..

[79] Langendonk R.F., Neill D.R., Fothergill J.L. (2021). The Building Blocks of Antimicrobial Resistance in Pseudomonas aeruginosa: Implications for Current Resistance-Breaking Therapies. Front. Cell. Infect. Microbiol..

[80] Tenover F.C. (2006). Mechanisms of Antimicrobial Resistance in Bacteria. Am. J. Med..

[81] Tsuchido T., Takano M. (1988). Senzitization by heat treatment of Escherichia coli K-12 cells to hydrophobic antibacterial compounds. Antimicrob. Agents Chemother..

[82] Blair J.M.A., Webber M.A., Baylay A.J., Ogbolu D.O., Piddock L.J.V. (2015). Molecular mechanisms of antibiotic resistance. Nat. Rev. Microbiol..

[83] Schroeder J.W., Yeesin P., Simmons L.A., Wang J.D. (2018). Sources of spontaneous mutagenesis in bacteria. Crit. Rev. Biochem. Mol. Biol..

[84] Barbier F., Luyt C.-E. (2016). Understanding resistance. Intensive Care Med..

[85] Ruppé É., Woerther P.L., Barbier F. (2015). Mechanisms of antimicrobial resistance in Gram-negative bacilli. Ann. Intensive Care.

[86] Craig M.R., Alvaro S.M. (2019). The evolution of antibiotic resistance. Science.

[87] Dubey G.P., Ben-Yehuda S. (2011). Intercellular Nanotubes Mediate Bacterial Communication. Cell.

[88] Domingues S., Nielsen K.M. (2017). Membrane vesicles and horizontal gene transfer in prokaryotes. Curr. Opin. Microbiol..

[89] Scharn C.R., Tenover F.C., Goering R.V. (2013). Transduction of staphylococcal cassette chromosome mec elements between strains of Staphylococcus aureus. Antimicrob. Agents Chemother..

[90] Dowson C.G., Coffey T.J., Kell C., Whiley R.A. (1993). Evolution of penicillin resistance in Streptococcus pneumoniae; the role of Streptococcus mitis in the formation of a low affinity PBP2B in S. pneumoniae. Mol. Microbiol..

[91] Unemo M., Golparian D., Nicholas R., Ohnishi M., Gallay A., Sednaouie P. (2012). High-level cefixime- and ceftriaxone-resistant Neisseria gonorrhoeae in France: Novel penA mosaic allele in a successful international clone causes treatment failure. Antimicrob. Agents Chemother..

[92] Frost L.S., Leplae R., Summers A.O., Toussaint A. (2005). Mobile genetic elements: The agents of open source evolution. Nat. Rev. Microbiol..

[93] Alekshun M.N., Levy S.B. (2007). Molecular Mechanisms of Antibacterial Multidrug Resistance. Cell.

[94] Paterson D.L., Bonomo R.A. (2005). Extended-spectrum β-lactamases: A clinical update. Clin. Microbiol. Rev..

[95] Kumarasamy K.K., Toleman M.A., Walsh T.R., Bagaria J., Butt F., Balakrishnan R., Chaudhary U., Doumith M., Giske C.G., Irfan S. (2010). Emergence of a new antibiotic resistance mechanism in India, Pakistan, and the UK: A molecular, biological, and epidemiological study. Lancet Infect. Dis..

[96] Yong D., Toleman M.A., Giske C.G., Cho H.S., Sundman K., Lee K., Walsh T.R. (2009). Characterization of a new metallo-β-lactamase gene, bla NDM-1, and a novel erythromycin esterase gene carried on a unique genetic structure in Klebsiella pneumoniae sequence type 14 from India. Antimicrob. Agents Chemother..

[97] Wozniak R.A.F., Waldor M.K. (2010). Integrative and conjugative elements: Mosaic mobile genetic elements enabling dynamic lateral gene flow. Nat. Rev. Microbiol..

[98] San Millan A. (2018). Evolution of Plasmid-Mediated Antibiotic Resistance in the Clinical Context. Trends Microbiol..

[99] Dijkshoorn L., Nemec A., Seifert H. (2007). An increasing threat in hospitals: Multidrug-resistant Acinetobacter baumannii. Nat. Rev. Microbiol..

[100] Levin A.S., Barone A.A., Penço J., Santos M.V., Marinho I.S., Arruda E.A.G., Manrique E.I., Costa S.F. (1999). Intravenous Colistin as Therapy for Nosocomial Infections Caused by Multidrug-Resistant Pseudomonas aeruginosa and Acinetobacter baumannii. Clin. Infect. Dis..

[101] Nikaido H. (2009). Multidrug resistance in bacteria. Annu. Rev. Biochem..

[102] Nikaido H. (1998). Multiple antibiotic resistance and efflux. Curr. Opin. Microbiol..

[103] Laurent P., Thierry L., Salih T., Esthel R., Jean-Louis G., Patrice N. (2001). Characterization of Class 1 Integrons from Pseudomonas aeruginosa That Contain the blaVIM-2Carbapenem-Hydrolyzing β-Lactamase Gene and of Two Novel Aminoglycoside Resistance Gene Cassettes. Antimicrob. Agents Chemother..

[104] Jacoby G.A., Medeiros A.A. (1991). More extended-spectrum β-lactamases. Antimicrob. Agents Chemother..

[105] Bonnet R. (2004). Growing Group of Extended-Spectrum β-Lactamases: The CTX-M Enzymes. Antimicrob. Agents Chemother..

[106] Drlica K., Malik M., Kerns R.J., Zhao X. (2008). Quinolone-mediated bacterial death. Antimicrob. Agents Chemother..

[107] Sharma S.K., Mohan A. (2006). Multidrug-resistant tuberculosis: A menace that threatens to destabilize tuberculosis control. Chest.

[108] Goldstein B.P. (2014). Resistance to rifampicin: A review. J. Antibiot..

[109] Alangaden G.J., Kreiswirth B.N., Aouad A., Khetarpal M., Igno F.R., Moghazeh S.L., Manavathu E.K., Lerner S.A. (1998). Mechanism of resistance to amikacin and kanamycin in Mycobacterium tuberculosis. Antimicrob. Agents Chemother..

[110] Zgurskaya H.I., López C.A., Gnanakaran S. (2016). Permeability Barrier of Gram-Negative Cell Envelopes and Approaches to Bypass It. ACS Infect. Dis..

[111] Baharoglu Z., Mazel D. (2011). Vibrio cholerae triggers SOS and mutagenesis in response to a wide range of antibiotics: A route towards multiresistance. Antimicrob. Agents Chemother..

[112] Foster P.L. (2007). Stress-induced mutagenesis in bacteria. Crit. Rev. Biochem. Mol. Biol..

[113] Miller C., Thomsen L.E., Gaggero C., Mosseri R., Ingmer H., Cohen S.N. (2004). SOS response induction by β-lactams and bacterial defense against antibiotic lethality. Science.

[114] Blázquez J., Couce A., Rodríguez-Beltrán J., Rodríguez-Rojas A. (2012). Antimicrobials as promoters of genetic variation. Curr. Opin. Microbiol..

[115] Galhardo R.S., Hastings P.J., Rosenberg S.M. (2007). Mutation as a stress response and the regulation of evolvability. Crit. Rev. Biochem. Mol. Biol..

[116] Oliver A., Cantón R., Campo P., Baquero F., Blázquez J. (2000). High frequency of hypermutable Pseudomonas aeruginosa in cystic fibrosis lung infection. Science.

[117] Diaz-Diaz S., Recacha E., Machuca J., García-Duque A., Docobo-Pérez F., Blázquez J., Pascual A., Rodríguez-Martínez J.M. (2021). Synergistic quinolone sensitization by targeting recA SOS response gene and oxidative stress. Antimicrob. Agents Chemother..

[118] Ciofu O., Mandsberg L.F., Bjarnsholt T., Wassermann T., Høiby N. (2010). Genetic adaptation of pseudomonas aeruginosa during chronic lung infection of patients with cystic fibrosis: Strong and weak mutators with heterogeneous genetic backgrounds emerge in mucA and/or lasR mutants. Microbiology.

[119] Garibyan L., Huang T., Kim M., Wolff E., Nguyen A., Nguyen T., Diep A., Hu K., Iverson A., Yang H. (2003). Use of the rpoB gene to determine the specificity of base substitution mutations on the Escherichia coli chromosome. DNA Repair.

[120] LeClerc J.E., Li B., Payne W.L., Cebula T.A. (1996). High mutation frequencies among Escherichia coli and Salmonella pathogens. Science.

[121] Gogarten J.P., Townsend J.P. (2005). Horizontal gene transfer, genome innovation and evolution. Nat. Rev. Microbiol..

[122] Schönknecht G., Chen W.H., Ternes C.M., Barbier G.G., Shrestha R.P., Stanke M., Bräutigam A., Baker B.J., Banfield J.F., Garavito R.M. (2013). Gene Transfer from Bacteria and Archaea Facilitated Evolution of an Extremophilic Eukaryote. Science.

[123] Lin M., Kussell E. (2019). Inferring bacterial recombination rates from large-scale sequencing datasets. Nat. Methods.

[124] Niehus R., Mitri S., Fletcher A.G., Foster K.R. (2015). Migration and horizontal gene transfer divide microbial genomes into multiple niches. Nat. Commun..

[125] Soucy S.M., Huang J., Gogarten J.P. (2015). Horizontal gene transfer: Building the web of life. Nat. Rev. Genet..

[126] Iwasaki W., Takagi T. (2009). Rapid Pathway Evolution Facilitated by Horizontal Gene Transfers across Prokaryotic Lineages. PLoS Genet..

[127] Gal-Mor O., Finlay B.B. (2006). Pathogenicity islands: A molecular toolbox for bacterial virulence. Cell. Microbiol..

[128] Power J.J., Pinheiro F., Pompei S., Kovacova V., Yüksel M., Rathmann I., Förster M., Lässig M., Maier B. (2021). Adaptive evolution of hybrid bacteria by horizontal gene transfer. Proc. Natl. Acad. Sci. USA.

[129] Frazão N., Sousa A., Lässig M., Gordo I. (2019). Horizontal gene transfer overrides mutation in *Escherichia coli* colonizing the mammalian gut. Proc. Natl. Acad. Sci. USA.

[130] Dagan T., Martin W. (2006). The tree of one percent. Genome Biol..

[131] Woods L.C., Gorrell R.J., Taylor F., Connallon T., Kwok T., McDonald M.J. (2020). Horizontal gene transfer potentiates adaptation by reducing selective constraints on the spread of genetic variation. Proc. Natl. Acad. Sci. USA.

[132] Perron G.G., Lee A.E.G., Wang Y., Huang W.E., Barraclough T.G. (2012). Bacterial recombination promotes the evolution of multi-drug-resistance in functionally diverse populations. Proc. R. Soc. B Biol. Sci..

[133] Cooper T.F. (2007). Recombination speeds adaptation by reducing competition between beneficial mutations in populations of Escherichia coli. PLoS Biol..

[134] Lorenz M.G., Wackernagel W. (1994). Bacterial gene transfer by natural genetic transformation in the environment. Microbiol. Rev..

[135] Bubendorfer S., Krebes J., Yang I., Hage E., Schulz T.F., Bahlawane C., Didelot X., Suerbaum S. (2016). Genome-wide analysis of chromosomal import patterns after natural transformation of Helicobacter pylori. Nat. Commun..

[136] Muller H.J. (1964). The relation of recombination to mutational advance. Mutat. Res. Mol. Mech. Mutagen..

[137] Good B.H., McDonald M.J., Barrick J.E., Lenski R.E., Desai M.M. (2017). The dynamics of molecular evolution over 60,000 generations. Nature.

[138] Narra H.P., Ochman H. (2006). Of What Use Is Sex to Bacteria?. Curr. Biol..

[139] Lerminiaux N.A., Cameron A.D.S. (2018). Horizontal transfer of antibiotic resistance genes in clinical environments. Can. J. Microbiol..

[140] Fournier P.E., Vallenet D., Barbe V., Audic S., Ogata H., Poirel L., Richet H., Robert C., Mangenot S., Abergel C. (2006). Comparative genomics of multidrug resistance in Acinetobacter baumannii. PLoS Genet..

[141] Hughes D., Andersson D.I. (2015). Evolutionary consequences of drug resistance: Shared principles across diverse targets and organisms. Nat. Rev. Genet..

[142] Brandis G., Wrande M., Liljas L., Hughes D. (2012). Fitness-compensatory mutations in rifampicin-resistant RNA polymerase. Mol. Microbiol..

[143] Huseby D.L., Pietsch F., Brandis G., Garoff L., Tegehall A., Hughes D. (2017). Mutation Supply and Relative Fitness Shape the Genotypes of Ciprofloxacin-Resistant Escherichia coli. Mol. Biol. Evol..

[144] Dunai A., Spohn R., Farkas Z., Lázár V., Györkei Á., Apjok G., Boross G., Szappanos B., Grézal G., Faragó A. (2019). Rapid decline of bacterial drug-resistance in an antibiotic-free environment through phenotypic reversion. Elife.

[145] Shcherbakov D., Akbergenov R., Matt T., Sander P., Andersson D.I., Böttger E.C. (2010). Directed mutagenesis of mycobacterium smegmatis 16S rRNA to reconstruct the in vivo evolution of aminoglycoside resistance in mycobacterium tuberculosis. Mol. Microbiol..

[146] Lipsitch M., Bergstrom C.T., Levin B.R. (2000). The epidemiology of antibiotic resistance in hospitals: Paradoxes and prescriptions. Proc. Natl. Acad. Sci. USA.

[147] Drlica K., Zhao X. (2007). Mutant selection window hypothesis updated. Clin. Infect. Dis..

[148] Gullberg E., Cao S., Berg O.G., Ilbäck C., Sandegren L., Hughes D., Andersson D.I. (2011). Selection of resistant bacteria at very low antibiotic concentrations. PLoS Pathog..

[149] Stanton I.C., Murray A.K., Zhang L., Snape J., Gaze W.H. (2020). Evolution of antibiotic resistance at low antibiotic concentrations including selection below the minimal selective concentration. Commun. Biol..

[150] Wistrand-Yuen E., Knopp M., Hjort K., Koskiniemi S., Berg O.G., Andersson D.I. (2018). Evolution of high-level resistance during low-level antibiotic exposure. Nat. Commun..

[151] Andersson D.I., Hughes D. (2014). Microbiological effects of sublethal levels of antibiotics. Nat. Rev. Microbiol..

[152] Oz T., Guvenek A., Yildiz S., Karaboga E., Tamer Y.T., Mumcuyan N., Ozan V.B., Senturk G.H., Cokol M., Yeh P. (2014). Strength of selection pressure is an important parameter contributing to the complexity of antibiotic resistance evolution. Mol. Biol. Evol..

[153] Markussen T., Marvig R.L., Gómez-Lozano M., Aanæs K., Burleigh A.E., Høiby N., Johansen H.K., Molin S., Jelsbak L. (2014). Environmental heterogeneity drives within-host diversification and evolution of Pseudomonas aeruginosa. MBio.

[154] Gerrish P.J., Lenski R.E. (1998). The fate of competing beneficial mutations in an asexual population. Genetica.

[155] Gifford D.R., MacLean R.C. (2013). Evolutionary reversals of antibiotic resistance in experimental populations of pseudomonas aeruginosa. Evolution.

[156] Qi Q., Toll-Riera M., Heilbron K., Preston G.M., Maclean R.C. (2016). The genomic basis of adaptation to the fitness cost of rifampicin resistance in pseudomonas aeruginosa. Proc. R. Soc. B Biol. Sci..

[157] Wong A. (2017). Epistasis and the evolution of antimicrobial resistance. Front. Microbiol..

[158] Angst D.C., Hall A.R. (2013). The cost of antibiotic resistance depends on evolutionary history in Escherichia coli. BMC Evol. Biol..

[159] Weinreich D.M., Delaney N.F., DePristo M.A., Hartl D.L. (2006). Darwinian evolution can follow only very few mutational paths to fitter proteins. Science.

[160] Schenk M.F., Szendro I.G., Krug J., de Visser J.A.G.M. (2012). Quantifying the adaptive potential of an antibiotic resistance enzyme. PLoS Genet..

[161] Rodríguez-Beltrán J., DelaFuente J., León-Sampedro R., MacLean R.C., San Millán Á. (2021). Beyond horizontal gene transfer: The role of plasmids in bacterial evolution. Nat. Rev. Microbiol..

[162] Trindade S., Sousa A., Xavier K.B., Dionisio F., Ferreira M.G., Gordo I. (2009). Positive epistasis drives the acquisition of multidrug resistance. PLoS Genet..

[163] Durão P., Trindade S., Sousa A., Gordo I. (2015). Multiple resistance at no cost: Rifampicin and streptomycin a dangerous liaison in the spread of antibiotic resistance. Mol. Biol. Evol..

[164] Levin-Reisman I., Brauner A., Ronin I., Balaban N.Q. (2019). Epistasis between antibiotic tolerance, persistence, and resistance mutations. Proc. Natl. Acad. Sci. USA.

[165] Lukačišinová M., Fernando B., Bollenbach T. (2020). Highly parallel lab evolution reveals that epistasis can curb the evolution of antibiotic resistance. Nat. Commun..

[166] Kryazhimskiy S., Rice D.P., Jerison E.R., Desai M.M. (2014). Global epistasis makes adaptation predictable despite sequence-level stochasticity. Science.

[167] MacLean R.C., Perron G.G., Gardner A. (2010). Diminishing Returns From Beneficial Mutations and Pervasive Epistasis Shape the Fitness Landscape for Rifampicin Resistance in Pseudomonas aeruginosa. Genetics.

[168] Andersson D.I., Levin B.R. (1999). The biological cost of antibiotic resistance. Curr. Opin. Microbiol..

[169] Maisnier-Patin S., Berg O.G., Liljas L., Andersson D.I. (2002). Compensatory adaptation to the deleterious effect of antibiotic resistance in Salmonella typhimurium. Mol. Microbiol..

[170] Nagaev I., Björkman J., Andersson D.I., Hughes D. (2001). Biological cost and compensatory evolution in fusidic acid-resistant Staphylococcus aureus. Mol. Microbiol..

[171] Comas I., Borrell S., Roetzer A., Rose G., Malla B., Kato-Maeda M., Galagan J., Niemann S., Gagneux S. (2012). Whole-genome sequencing of rifampicin-resistant Mycobacterium tuberculosis strains identifies compensatory mutations in RNA polymerase genes. Nat. Genet..

[172] Moura de Sousa J., Balbontín R., Durão P., Gordo I. (2017). Multidrug-resistant bacteria compensate for the epistasis between resistances. PLoS Biol..

[173] Baym M., Stone L.K., Kishony R. (2016). Multidrug evolutionary strategies to reverse antibiotic resistance. Science.

[174] Chait R., Craney A., Kishony R. (2007). Antibiotic interactions that select against resistance. Nature.

[175] Michel J.B., Yeh P.J., Chait R., Moellering R.C., Kishony R. (2008). Drug interactions modulate the potential for evolution of resistance. Proc. Natl. Acad. Sci. USA.

[176] Imamovic L., Sommer M.O.A. (2013). Use of collateral sensitivity networks to design drug cycling protocols that avoid resistance development. Sci. Transl. Med..

[177] Lázár V., Nagy I., Spohn R., Csörgo B., Györkei Á., Nyerges Á., Horváth B., Vörös A., Busa-Fekete R., Hrtyan M. (2014). Genome-wide analysis captures the determinants of the antibiotic cross-resistance interaction network. Nat. Commun..

[178] Barbosa C., Beardmore R., Schulenburg H., Jansen G. (2018). Antibiotic combination efficacy (ACE) networks for a Pseudomonas aeruginosa model. PLoS Biol..

[179] Rosenkilde C.E.H., Munck C., Porse A., Linkevicius M., Andersson D.I., Sommer M.O.A. (2019). Collateral sensitivity constrains resistance evolution of the CTX-M-15 β-lactamase. Nat. Commun..

[180] Roemhild R., Andersson D.I. (2021). Mechanisms and therapeutic potential of collateral sensitivity to antibiotics. PLoS Pathog..

[181] Yen P., Papin J.A. (2017). History of antibiotic adaptation influences microbial evolutionary dynamics during subsequent treatment. PLOS Biol..

[182] Nichol D., Rutter J., Bryant C., Hujer A.M., Lek S., Adams M.D., Jeavons P., Anderson A.R.A., Bonomo R.A., Scott J.G. (2019). Antibiotic collateral sensitivity is contingent on the repeatability of evolution. Nat. Commun..

[183] Barbosa C., Römhild R., Rosenstiel P., Schulenburg H. (2019). Evolutionary stability of collateral sensitivity to antibiotics in the model pathogen Pseudomonas aeruginosa. Elife.

[184] Hughes D., Andersson D.I. (2017). Evolutionary Trajectories to Antibiotic Resistance. Annu. Rev. Microbiol..

[185] Garoff L., Pietsch F., Huseby D.L., Lilja T., Brandis G., Hughes D. (2020). Population Bottlenecks Strongly Influence the Evolutionary Trajectory to Fluoroquinolone Resistance in Escherichia coli. Mol. Biol. Evol..

[186] Vogwill T., Phillips R.L., Gifford D.R., Maclean R.C. (2016). Divergent evolution peaks under intermediate population bottlenecks during bacterial experimental evolution. Proc. R. Soc. B Biol. Sci..

